# Effects of Variety and Growing Location on Physicochemical Properties of Starch from Sweet Potato Root Tuber

**DOI:** 10.3390/molecules26237137

**Published:** 2021-11-25

**Authors:** Laiquan Shi, Yibo Li, Lingshang Lin, Xiaofeng Bian, Cunxu Wei

**Affiliations:** 1 Key Laboratory of Crop Genetics and Physiology of Jiangsu Province/Jiangsu Key Laboratory of Crop Genomics and Molecular Breeding, Yangzhou University, Yangzhou 225009, China; MZ120201523@yzu.edu.cn (L.S.); DX120180133@yzu.edu.cn (Y.L.); 007520@yzu.edu.cn (L.L.); 2 Co-Innovation Center for Modern Production Technology of Grain Crops of Jiangsu Province/Joint International Research Laboratory of Agriculture & Agri-Product Safety of the Ministry of Education, Yangzhou University, Yangzhou 225009, China; 3 Institute of Food Crops, Jiangsu Academy of Agricultural Sciences, Nanjing 210014, China; bianxiaofeng2@163.com

**Keywords:** sweet potato, variety, growing location, starch, physicochemical properties

## Abstract

Three sweet potato varieties with purple-, yellow-, and white-fleshed root tubers were planted in four growing locations. Starches were isolated from their root tubers, their physicochemical properties (size, iodine absorption, amylose content, crystalline structure, ordered degree, lamellar thickness, swelling power, water solubility, and pasting, thermal and digestion properties) were determined to investigate the effects of variety and growing location on starch properties in sweet potato. The results showed that granule size (D[4,3]) ranged from 12.1 to 18.2 μm, the iodine absorption parameters varied from 0.260 to 0.361 for OD620, from 0.243 to 0.326 for OD680 and from 1.128 to 1.252 for OD620/550, and amylose content varied from 16.4% to 21.2% among starches from three varieties and four growing locations. Starches exhibited C-type X-ray diffraction patterns, and had ordered degrees from 0.634 to 0.726 and lamellar thicknesses from 9.72 to 10.21 nm. Starches had significantly different swelling powers, water solubilities, pasting viscosities, and thermal properties. Native starches had rapidly digestible starch (RDS) from 2.2% to 10.9% and resistant starch (RS) from 58.2% to 89.1%, and gelatinized starches had RDS from 70.5% to 81.4% and RS from 10.8% to 23.3%. Two-way ANOVA analysis showed that starch physicochemical properties were affected significantly by variety, growing location, and their interaction in sweet potato.

## 1. Introduction

Sweet potato (*Ipomoea batatas*), an essential root tuber starchy crop, is widely cultivated in over 100 countries. There are over 90 million tonnes in annual production [1]. The root tuber contains about 50–80% starch in dry basis (d.b.) [2,3]. Sweet potato starch is widely used to make conventional noodle, vermicelli, thickening agent, and syrup in processed foods, and can also produce film, ethanol, citric acid, lactic acid, and other chemicals in non-food industries [4,5]. The physicochemical properties of starch determine its utilizations in food and nonfood industries [3]. Therefore, the research of sweet potato starch has been getting great attentions.

Due to artificial selection, natural hybrids, and mutations, there are hundreds of varieties or lines of sweet potato [4]. Their root tubers have different flesh colors [2,6]. Starches from different varieties have been investigated for their physicochemical properties and utilizations in sweet potato [2,3,7,8,9]. Though starch content in root tuber exhibits significant differences among different colored varieties, starch properties are not affected by the color of root tuber [2,8].

Effects of plant growing condition on starch properties have been reported in sweet potato [10,11,12,13]. Crystalline structure of starch significantly changes when sweet potato is planted in soils with different temperatures. A-type starch forms and accumulates in root tuber grown in soil with high temperature, and B-type crystallinity gradually does in root tuber with the decrease of soil temperature [11]. Recently, Duan et al. [10] planted one sweet potato variety in soils with different treatments of nitrogen fertilizer, and found that nitrogen treatment can change starch physicochemical properties. However, Noda et al. [13] found that starch properties do not significantly change in four sweet potato varieties grown in different levels of fertilizer. Guo et al. [12] planted three white, three yellow, and three purple fleshed varieties in soils with three treatments of nitrogen fertilizer, and found that nitrogen treatments have different effects on starch properties among different varieties, indicating that different genotypes have different responses to nitrogen fertilizer treatment.

In recent years, the effects of genotype and growing environment on starch characteristics have been studied in rice [14,15], wheat [16], potato [17,18], and cassava [19]. The effects exhibit some differences among different references or crops. For examples, Li and Liu [14] reported that components and chain-length distribution of rice starch are affected significantly by variety, growing location, and their interaction, but Sar et al. [15] found that the structures of amylose and amylopectin are affected only by varieties but have no significant relationship with growing location and the interaction of variety and growing location. Pelpolage et al. [18] reported that thermal properties and pasting peak, breakdown and final viscosities of potato starch are significantly affected by variety, growing location, and their interaction, but Ahmed et al. [17] found that gelatinization conclusion temperature, gelatinization enthalpy, and pasting final viscosity of potato starch are not affected by growing location. In addition, Nhan and Copeland [16] reported that thermal and pasting properties of wheat starch are affected significantly by variety and growing location. For cassava starch, some physicochemical properties are affected significantly by variety, or growing location, or both variety and growing location, or the interaction of variety and growing location [19]. Sweet potato is extensively grown in the tropical and subtropical areas [6]. However, the effects of growing location on starch characteristics are unclear in sweet potato, restricting the utilization of sweet potato variety in different regions.

Therefore, the objective of this work was to study the effect of sweet potato variety and growing location on the changes of starch properties. To achieve this objective, three sweet potato varieties with purple-, yellow-, and white-fleshed root tubers planted in four different growing locations were collected and analyzed their physicochemical properties (size, iodine absorption, amylose content, crystalline structure, ordered degree, lamellar thickness, swelling power, water solubility, and pasting, thermal and digestion properties).

## 2. Results and Discussion

### 2.1. Effects of Variety and Growing Location on Starch Size

**Figure 1:**
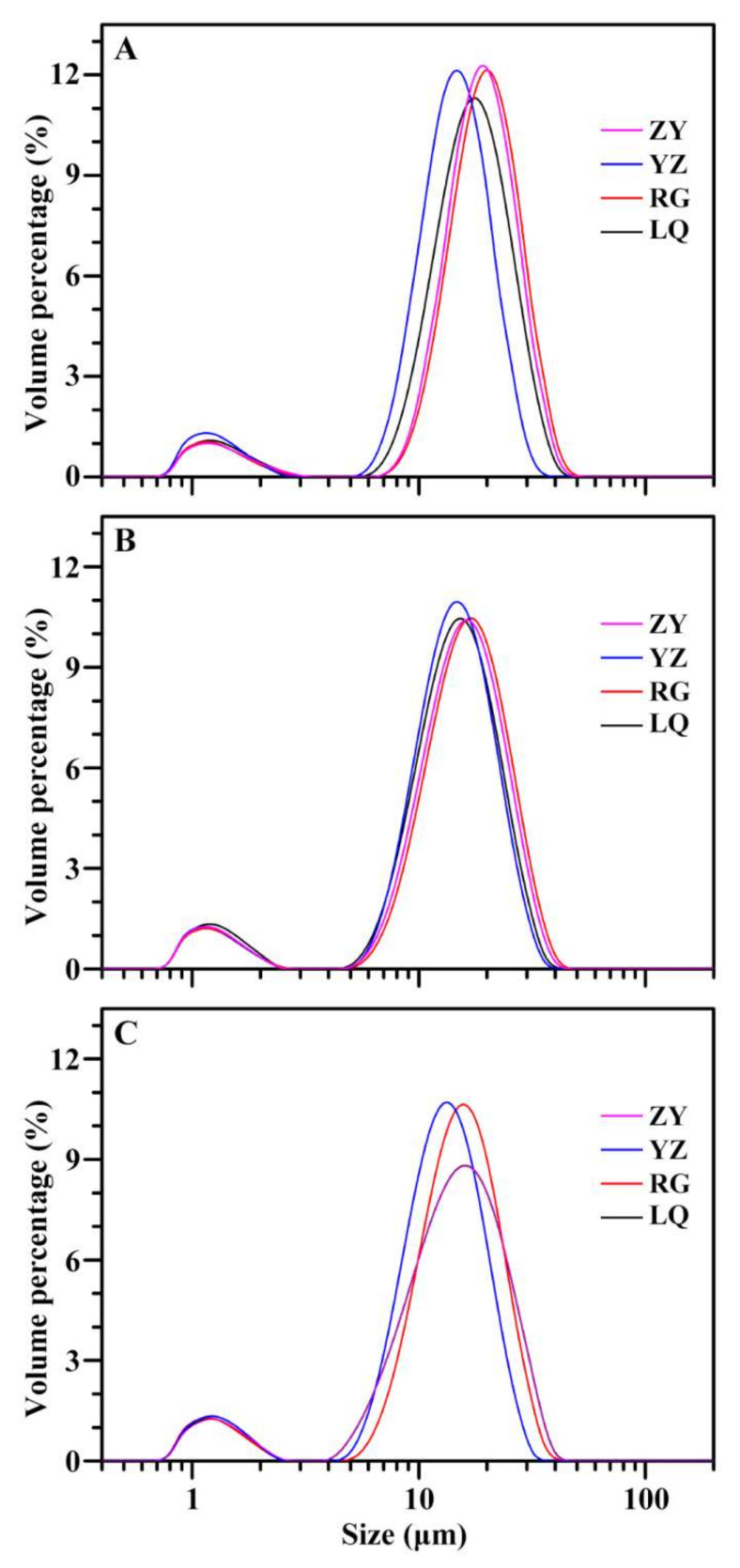
Granule size distributions of starches from root tubers of Ningzishu 1 (**A**), Sushu 16 (**B**), and Sushu 28 (**C**) in growing locations of Linquan (LQ), Rugao (RG), Yangzhou (YZ), and Zunyi (ZY).

Starches from three varieties in four growing locations followed similar size distribution patterns (Figure 1). Though there were two granule populations about from 0.8 to 3 μm and from 6 to 40 μm, the small-sized granules had very low weight percentage. Similar phenomenon has been reported in previous papers [2,9]. In the present research, the volume-weighted mean diameter (D[4,3]) was used to reflect the granule size (Table 1). Among four growing locations, granule size varied from 13.33 μm (YZ) to 18.22 μm (RG), from 13.53 μm (YZ) to 15.39 μm (RG), and from 12.11 μm (YZ) to 14.19 μm (RG) for Ningzishu 1, Sushu 16, and Sushu 28, respectively. Three sweet potato varieties all exhibited the largest starch size in growing location of RG and the smallest starch size in growing location of YZ. Two-way ANOVA analysis showed that starch size was significantly affected by variety, growing location, and their interaction (*p* < 0.001) (Table 1). Abegunde et al. [20] found that starch size is affected by plant physiology except variety and growing condition in sweet potato. Similar conclusion is also given in potato [18]. Guo et al. [2] reported that starches have significantly different granule sizes among different colored sweet potato varieties grown in the same environment, and the starch size is determined mainly by genotype but has no relationship with fleshed color.

### 2.2. Effects of Variety and Growing Location on Starch–Iodine Absorption Parameters

**Figure 2:**
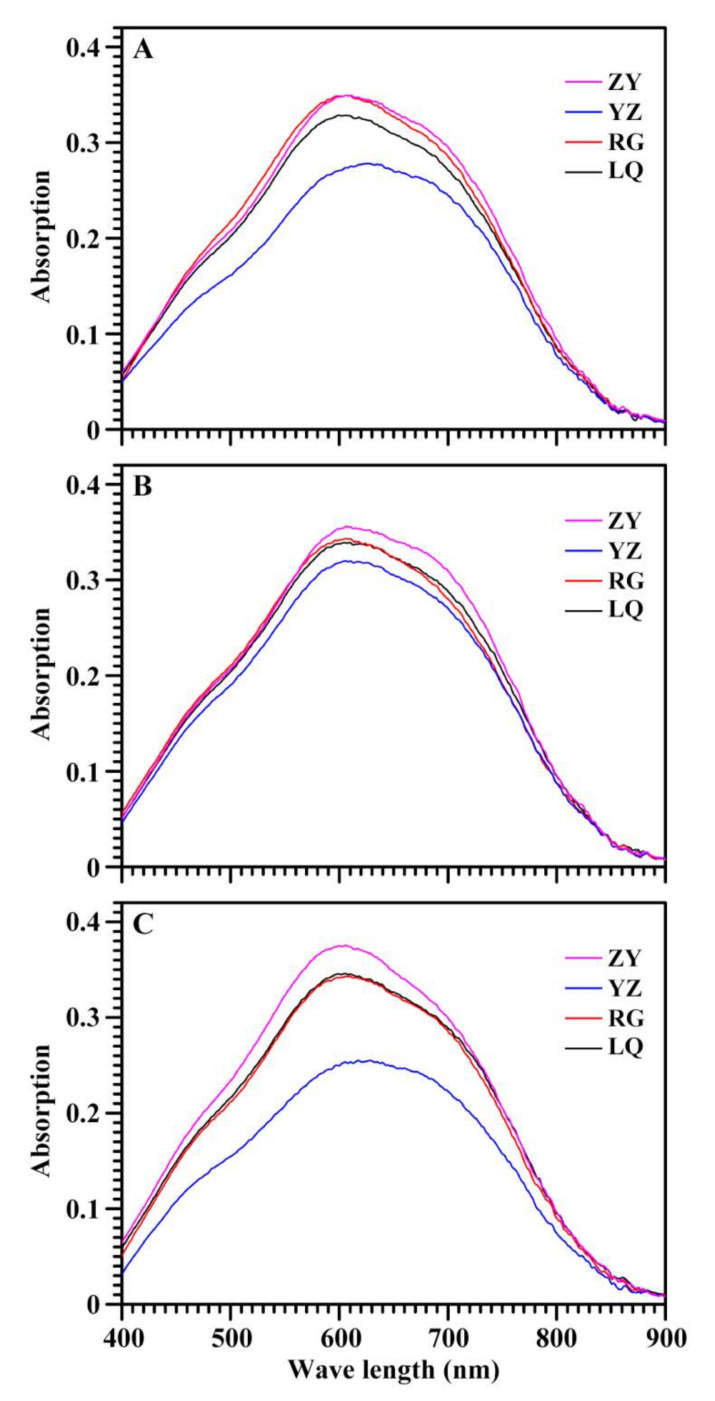
Iodine absorption spectra of starches from root tubers of Ningzishu 1 (**A**), Sushu 16 (**B**), and Sushu 28 (**C**) in growing locations of Linquan (LQ), Rugao (RG), Yangzhou (YZ), and Zunyi (ZY).

The starch–iodine absorption spectrum is presented in Figure 2, and spectrum parameters including OD620, OD680, OD620/550 and the maximum absorption wavelength (λmax) are presented in Table 1. The OD620 indicates the iodine-binding of amylose and amylopectin long branch-chains, and the OD680 (also called blue value) reflects the iodine-binding capacity of starch [21]. The OD620 of starch–iodine complex ranged from 0.275 (YZ) to 0.361 (ZY), from 0.322 (YZ) to 0.353 (ZY), and from 0.260 (YZ) to 0.347 (ZY) for Ningzishu 1, Sushu 16, and Sushu 28, respectively. The OD680 ranged from 0.257 (YZ) to 0.321 (ZY), from 0.294 (YZ) to 0.326 (ZY), and from 0.243 (YZ) to 0.316 (ZY) for Ningzishu 1, Sushu 16, and Sushu 28, respectively, indicating that sweet potato grown in YZ and ZY had the lowest and the highest starch–iodine binding ability, respectively. The OD620/550, absorption ratio of OD620 and OD550, is an index reflecting the relative proportion of long molecule chains in starch [21]. The OD620/550 varied from 1.128 (ZY) to 1.232 (YZ), from 1.193 (RG) to 1.235 (ZY), and from 1.175 (LQ) to 1.252 (YZ) for Ningzishu 1, Sushu 16, and Sushu 28, respectively. The λmax among four growing locations varied from 606.1 nm (RG) to 625.6 nm (YZ), from 609.2 nm (YZ) to 613.9 nm (LQ), and from 606.4 nm (ZY) to 629.0 nm (YZ) for Ningzishu 1, Sushu 16, and Sushu 28, respectively. Two-way ANOVA analysis showed that starch–iodine absorption parameters of OD620, OD680, OD620/550 and λmax were significantly affected by variety, growing location, and their interaction (*p* < 0.001) (Table 1). Both amylose/amylopectin ratio and amylopectin structure determine the starch–iodine absorption parameters [22]. Starch is synthesized by some starch synthesis-related enzymes. The expression regulation of these enzymes is influenced by genotype and growing condition of plant, especially for environment temperature [23,24,25].

### 2.3. Effects of Variety and Growing Location on Amylose Content of Starch

**Figure 3:**
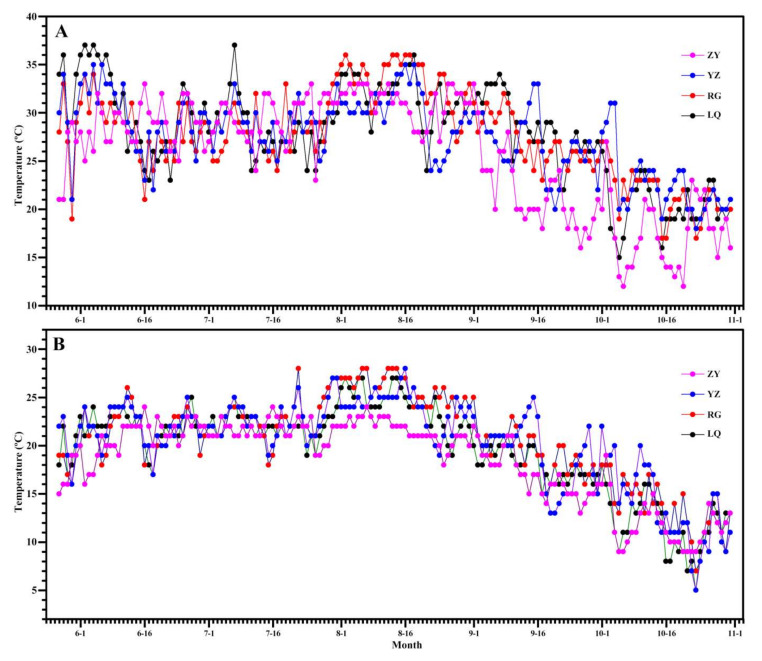
High temperature (**A**) and low temperature (**B**) of every day during growth stage of sweet potato in growing locations of Linquan (LQ), Rugao (RG), Yangzhou (YZ), and Zunyi (ZY).

The AC determines starch physicochemical properties, and can be measured with iodine colorimetry and concanavalin A precipitation [21]. The iodine colorimetry is often used to evaluate the apparent amylose content due to that iodine can bind amylopectin long branch-chains to overestimate AC. The concanavalin A can specifically bind with amylopectin and form precipitation. The ratio of amylose to both amylose and amylopectin measured by concanavalin A precipitation is usually called true amylose content due to being not influenced by starch purity and amylopectin long branch-chains [21]. In this study, the AC was measured using concanavalin A precipitation method (Table 1). The AC ranged from 16.4% (LQ) to 17.7% (ZY), from 18.4% (LQ) to 21.2% (ZY), and from 18.3% (YZ) to 21.1% (RG) for Ningzishu 1, Sushu 16, and Sushu 28, respectively. Two-way ANOVA analysis showed that AC was significantly affected by variety, growing location, and their interaction (Table 1). Some researchers reported that both genotype and environment contribute significantly to the variability in AC in wheat [26] and rice [14]. Amylose is synthesized by granule-bound starch synthase I (GBSSI) encoded by *Waxy* (*Wx*) in plant storage tissue. Many *Wx* alleles have been reported in cereal crops. They are responsible for significantly different expressions and activities of GBSSI, leading to starches with different ACs among different varieties [27]. Therefore, it is easy to understand that amylose is determined by genotype. Some references reported that AC has no significant effect from the growing location, and is influenced only by genotype in potato [18], cassava [19], and rice [15]. However, more references reported that *Wx* expression and GBSSI activity are influenced by environment temperature. The low temperature increases amylose synthesis, and high temperature decreases amylose synthesis [23,24]. In the present study, four growing locations had different temperatures during sweet potato starch development (Figure 3). Therefore, AC is affected by variety, growing location, and their interaction in sweet potato.

### 2.4. Effects of Variety and Growing Location on Crystalline Structure of Starch

**Figure 4:**
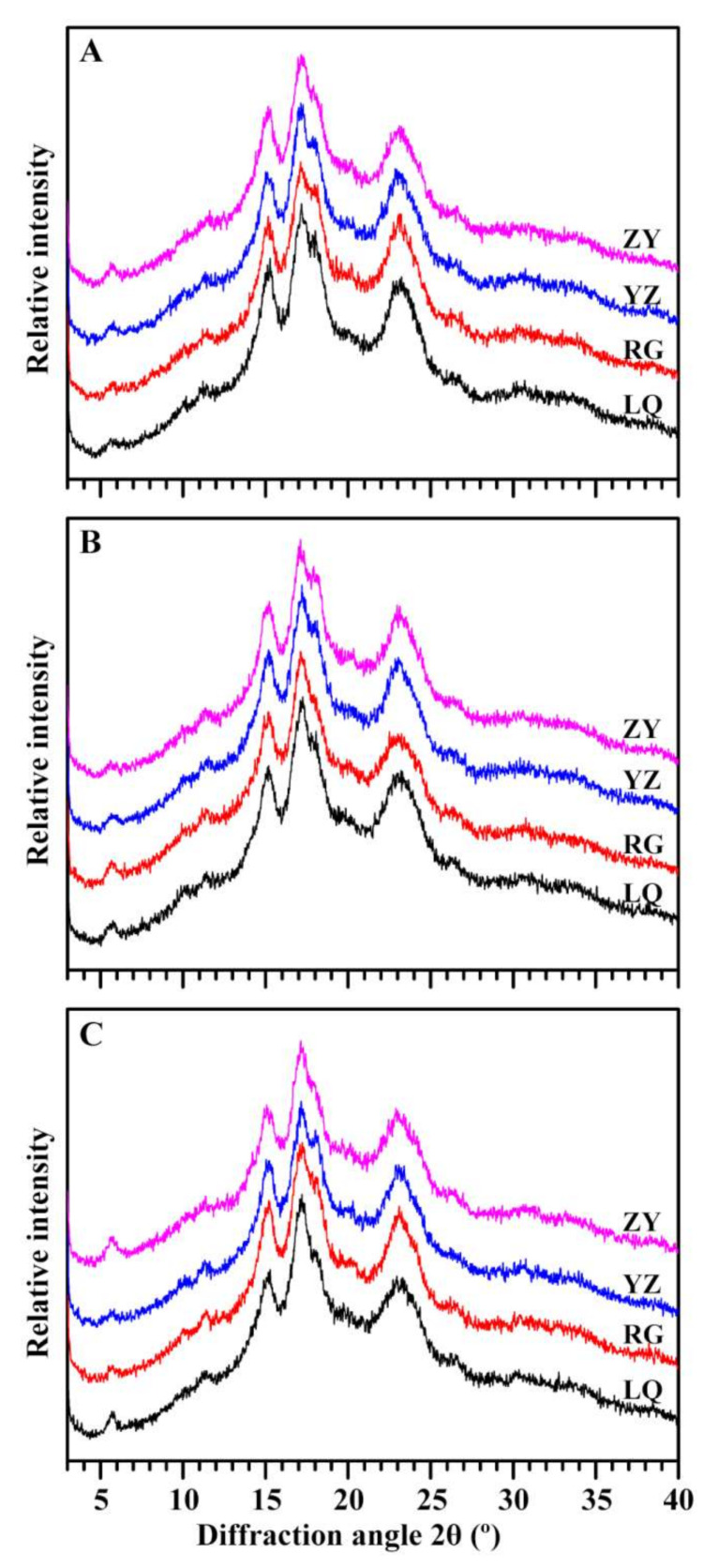
XRD spectra of starches from root tubers of Ningzishu 1 (**A**), Sushu 16 (**B**), and Sushu 28 (**C**) in growing locations of Linquan (LQ), Rugao (RG), Yangzhou (YZ), and Zunyi (ZY).

The XRD patterns of starches from three sweet potato varieties and four growing locations are shown in Figure 4. Amylopectin in plants can form A- and B-type crystallinities. Native starches from botany sources are usually divided into A-, B-, and C-type according to their crystallinity types [28]. A- and B-type starch contains only A- and B-type crystallinity, respectively, but C-type starch contains both A- and B-type crystallinities. According to the proportion of A- and B-type crystallinity from high to low, C-type starch is usually divided into C_A_-, C_C_-, and C_B_-type [28]. Though XRD patterns of starches from Ningzishu 1, Sushu 16, and Sushu 28 grown in LQ, RG, YZ, and ZY all exhibited C-type, the intensity of shoulder peak at 2θ 18°, a characteristic peak of A-type crystallinity, was significantly different, exhibiting a different proportion of A- and B-type crystallinity in starch (Figure 4). A-, C_A_-, C_C_-, and C_B_-type starches have been found in some sweet potato varieties [9,29,30]. Genkina et al. [11] found that A-type starch forms and accumulates in sweet potato root tuber grown in soil of high temperature (over 33 °C), and C_C_-type starch exists in root tuber grown in soil of low temperature about 15 °C. Guo et al. [31] found that A-, B-, and C-type starch granules can simultaneously exist in root tuber of sweet potato. Therefore, variety and environment temperature affect starch crystalline structure of sweet potato. However, RC of starch was found to have no significant relationship with sweet potato variety and growing location (*p* > 0.05) (Table 2).

### 2.5. Effects of Variety and Growing Location on Ordered Structure of Starch

**Figure 5:**
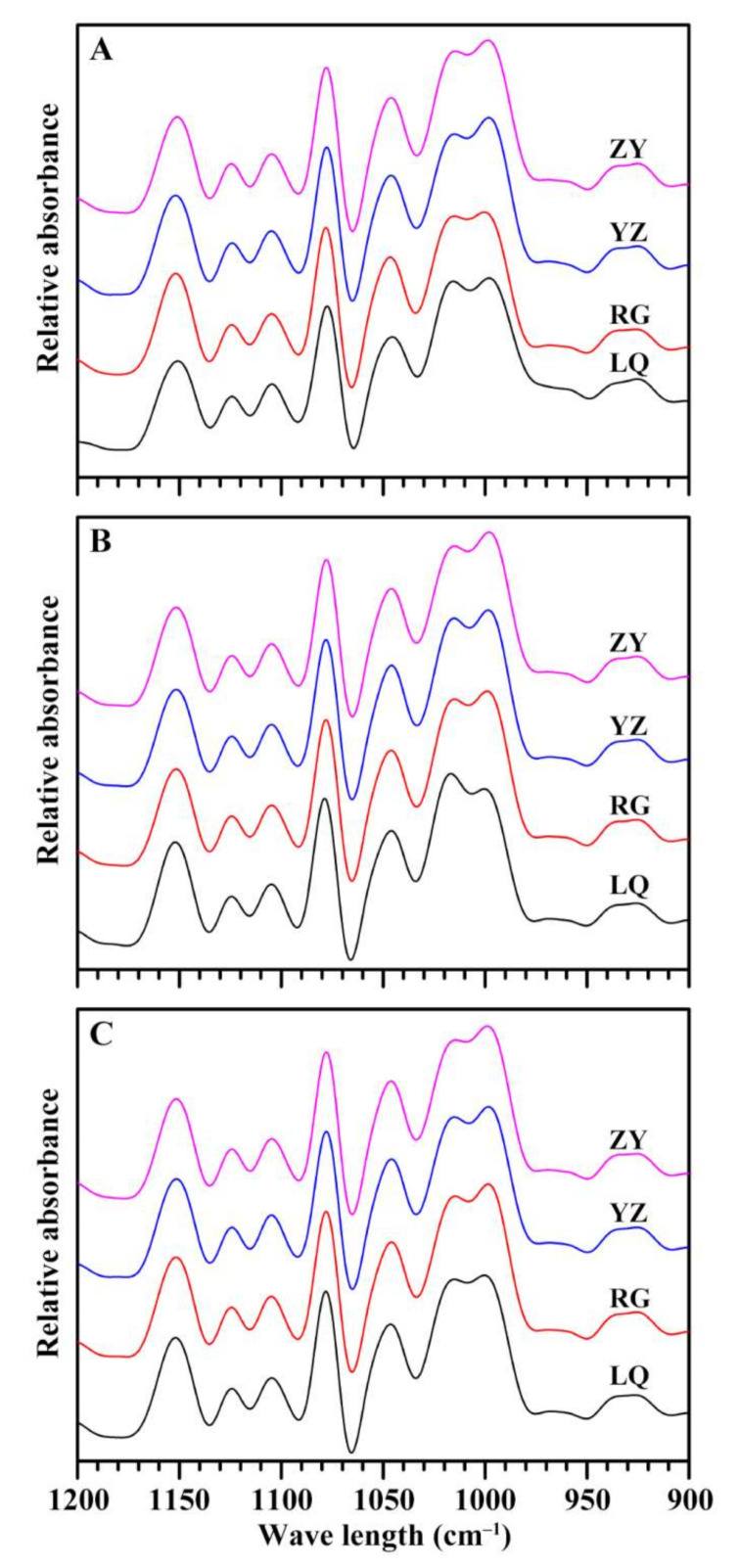
FTIR spectra of starches from root tubers of Ningzishu 1 (**A**), Sushu 16 (**B**), and Sushu 28 (**C**) in growing locations of Linquan (LQ), Rugao (RG), Yangzhou (YZ) and Zunyi (ZY).

The deconvolution FTIR spectra of starches from different varieties and growing locations are shown in Figure 5. The absorption bands at 1045 and 1022 cm^–1^ are associated with crystalline and amorphous regions in starch, respectively [32]. The intensity ratio of band at 1045 to 1022 cm^–1^ (1045/1022 cm^–1^) is often used as an index reflecting the ordered degree, and that at 1022 to 995 cm^–1^ (1022/995 cm^–1^) indicates the proportion of amorphous to ordered carbohydrate structure in starch [33]. The 1045/1022 cm^–1^ ranged from 0.650 (LQ) to 0.709 (YZ), from 0.634 (LQ) to 0.702 (YZ), and from 0.678 (LQ) to 0.726 (YZ) for Ningzishu 1, Sushu 16, and Sushu 28, respectively. The 1022/995 cm^–1^ varied from 0.896 (YZ) to 0.983 (RG), from 0.922 (ZY) to 1.103 (LQ), and from 0.911 (ZY) to 0.977 (LQ) for Ningzishu 1, Sushu 16, and Sushu 28, respectively. Two-way ANOVA analysis showed that ordered structure of sweet potato starch was significantly affected by variety, growing location, and their interaction (Table 2).

### 2.6. Effects of Variety and Growing Location on Lamellar Structure of Starch

**Figure 6:**
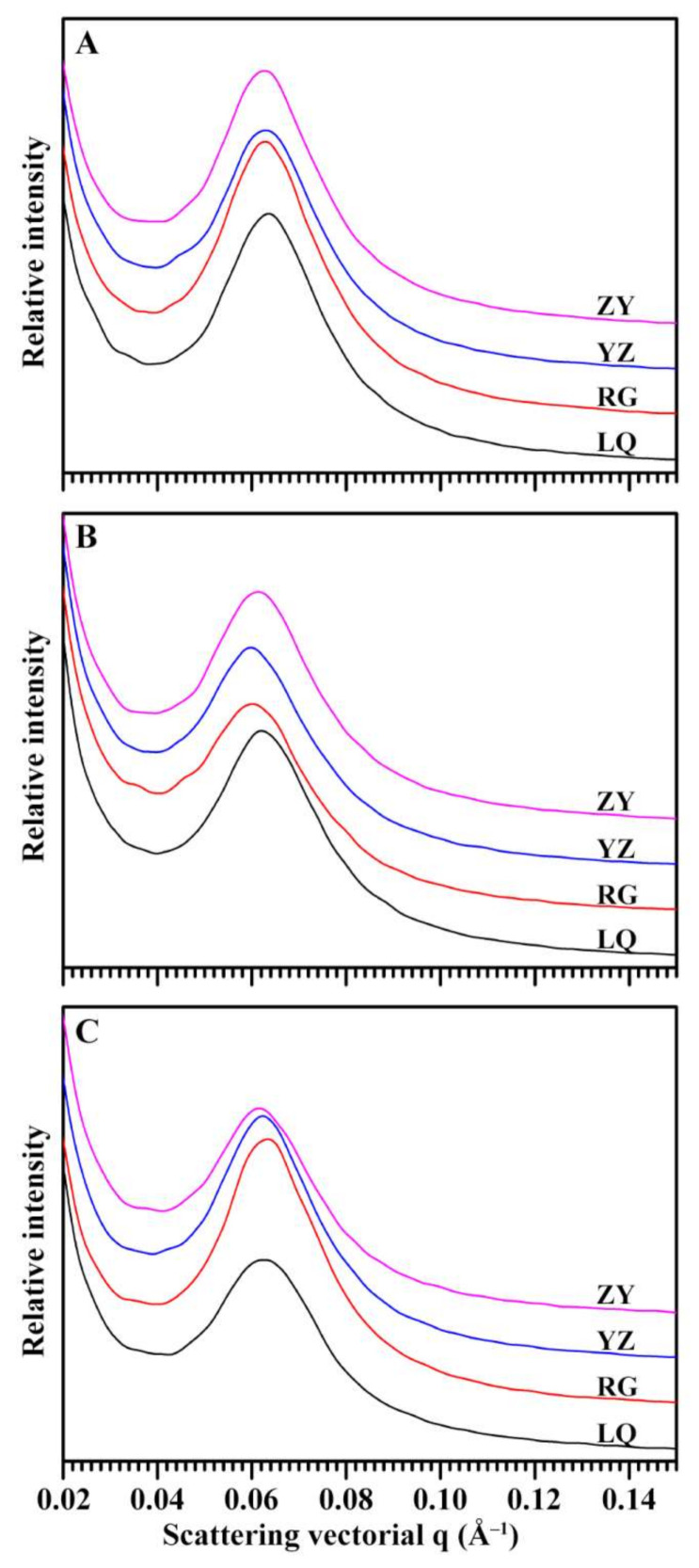
SAXS profiles of starches from root tubers of Ningzishu 1 (**A**), Sushu 16 (**B**), and Sushu 28 (**C**) in growing locations of Linquan (LQ), Rugao (RG), Yangzhou (YZ) and Zunyi (ZY).

The SAXS spectra of starches from different sweet potato varieties grown in different locations are presented in Figure 6. Their intensities at 0.2 Å^–1^ were normalized, resulting in comparable peak intensities among different samples [34]. The peak intensity of SAXS profile is determined by the ordered structure in semicrystalline regions, and lamellar distance reflects the thickness of repeated crystalline and amorphous lamellae [35]. The lamellar structure is formed by amylopectin branch-chains [36]. The peak intensity ranged from 357.1 (YZ) to 418.6 (RG), from 257.7 (RG) to 332.4 (ZY), and from 275.5 (LQ) to 417.8 (RG) for Ningzishu 1, Sushu 16, and Sushu 28, respectively. The lamellar thickness ranged from 9.72 nm (LQ) to 9.82 nm (ZY), from 9.86 nm (LQ) to 10.21 nm (YZ), and from 9.76 nm (RG) to 9.89 nm (ZY) for Ningzishu 1, Sushu 16, and Sushu 28, respectively. Two-way ANOVA analysis exhibited that lamellar peak intensity and thickness of sweet potato starch were significantly affected by variety, growing location, and their interaction (Table 2).

### 2.7. Effects of Variety and Growing Location on Pasting Properties of Starch

**Figure 7:**
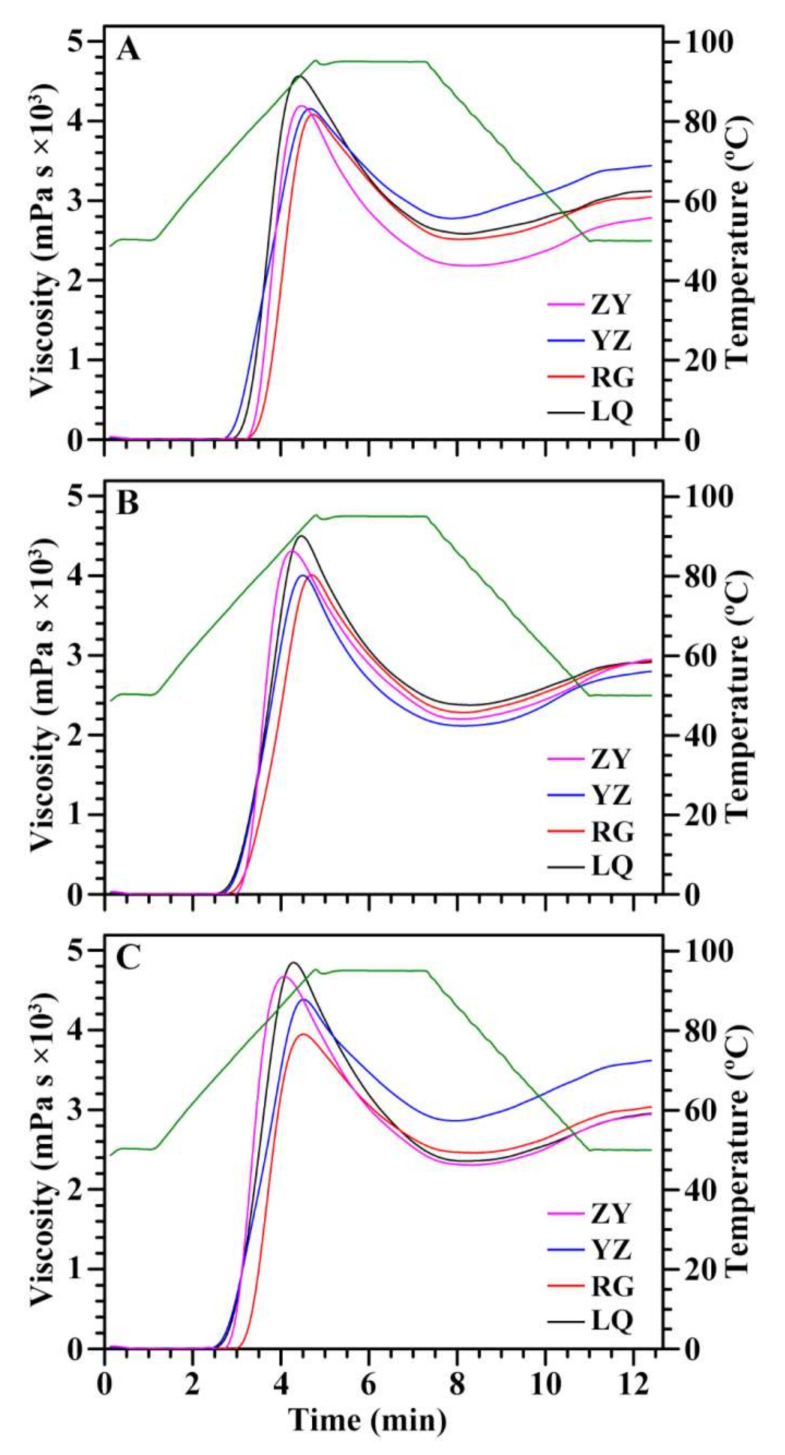
RVA profiles of starches from root tubers of Ningzishu 1 (**A**), Sushu 16 (**B**), and Sushu 28 (**C**) in growing locations of Linquan (LQ), Rugao (RG), Yangzhou (YZ), and Zunyi (ZY).

RVA can heat and cool an aqueous suspension of starch under constant shear and simultaneously measures the viscosity, producing a RVA curve of viscosity change as a function of temperature and time [37]. RVA curve is usually converted into RVA parameters including pasting temperature (P_Temp_), peak time (P_Time_), and peak (PV), hot (HV), breakdown (BV), final (FV), and setback viscosity (SV). These parameters are used as pasting properties of starch, which reflect eating, cooking, and processing qualities of starch and are also important parameters to design starch blend products as well as new products based on starch [19,37]. RVA curves of sweet potato starches from different varieties grown in different locations are shown in Figure 7. PV refers to the maximum viscosity during heating of starch paste, reflecting the capability of binding water and the granular swelling of starch [37]. PV ranged from 4171 mPa s (RG) to 4635 mPa s (LQ), from 4102 mPa s (YZ) to 4656 mPa s (LQ), and from 3918 mPa s (RG) to 4929 mPa s (LQ) for Ningzishu 1, Sushu 16, and Sushu 28, respectively (Table 3). The swollen granules disrupt during heating, leading to the decrease of viscosity. The HV is the lowest viscosity of heating starch paste, and is affected by granule swelling, amylose leaching, and amylose–lipid complex [38]. HV ranged from 2171 mPa s (ZY) to 2788 mPa s (YZ), from 2113 mPa s (YZ) to 2363 mPa s (LQ), and from 2274 mPa s (ZY) to 2846 mPa s (YZ) for Ningzishu 1, Sushu 16, and Sushu 28, respectively (Table 3). BV is the different value between PV and HV, reflecting paste resistance to heat and shear [20]. BV ranged from 1504 mPa s (YZ) to 2102 mPa s (ZY), from 1839 mPa s (RG) to 2293 mPa s (LQ), and from 1479 mPa s (RG) to 2591 mPa s (LQ) for Ningzishu 1, Sushu 16, and Sushu 28, respectively (Table 3). FV is the viscosity of starch paste after cooling, and indicates the stability to cooled starch paste or gel [2]. FV ranged from 2802 mPa s (ZY) to 3471 mPa s (YZ), from 2769 mPa s (YZ) to 2947 mPa s (ZY), and from 2920 mPa s (ZY) to 3579 mPa s (YZ) for Ningzishu 1, Sushu 16, and Sushu 28, respectively (Table 3). SB is the different viscosity between FV and HV, reflecting the tendency of starch pastes to retrograde [20]. SV ranged from 541 mPa s (LQ) to 682 mPa s (YZ), from 562 mPa s (LQ) to 769 mPa s (ZY), and from 587 mPa s (RG) to 733 mPa s (YZ) for Ningzishu 1, Sushu 16, and Sushu 28, respectively. P_Temp_ reflects the energy cost required during cooking, and ranged from 73.08 °C (YZ) to 78.98 °C (ZY), from 71.68 °C (LQ) to 76.05 °C (ZY), and from 70.07 °C (YZ) to 76.88 °C (RG) for Ningzishu 1, Sushu 16, and Sushu 28, respectively (Table 3). P_Time_ is the time of PV, and can reflect starch swelling with high swelling starch having low P_Time_ [20]. P_Time_ ranged from 4.40 min (LQ) to 4.62 min (YZ), from 4.22 min (ZY) to 4.60 min (RG), and from 4.07 min (ZY) to 4.51 (RG) for Ningzishu 1, Sushu 16, and Sushu 28, respectively. Two-way ANOVA analysis showed that the RVA parameters were significantly affected by variety, growing location, and their interaction (Table 3). Similar phenomenon exists in potato starch [18]. However, Ahmed et al. [17] reported that no significant effects of environment are observed on HV, FV, SV and P_Temp_ of potato starch. Nhan and Copeland [16] reported that PV, HV, and FV of wheat starch are affected by genotype, growing location, and their interaction, but BV, SV, and P_Temp_ are not affected by genotype. Tappiban et al. [19] reported that BV of cassava starch is affected by genotype, environment, and their interaction; PV is affected by genotype and environment; P_Temp_ and P_Time_ are affected by genotype; HV, FV, and SV are not affected by genotype, environment, and their interaction.

### 2.8. Effects of Variety and Growing Location on Swelling Power and Water Solubility of Starch

Swelling powers and water solubilities of sweet potato starches at 95 °C are presented in Table 4. Swelling power ranged from 29.9 g/g (RG) to 30.9 g/g (LQ), from 29.1 g/g (ZY) to 32.6 g/g (LQ), and from 26.7 g/g (YZ) to 29.8 g/g (ZY) for Ningzishu 1, Sushu 16, and Sushu 28, respectively. Water solubility ranged from 12.5% (RG) to 14.4% (ZY), from 14.2% (YZ) to 17.9% (ZY), and from 12.8% (YZ) to 15.3% (ZY) for Ningzishu 1, Sushu 16, and Sushu 28, respectively. The results are comparable with previous reports that swelling powers range from 24.5 to 32.7 g/g and water solubilities range from 12.1% to 24.1% among starches from 44 sweet potato varieties [39]. Two-way ANOVA analysis indicated that swelling power and water solubility of sweet potato starch were significantly affected by variety, growing location, and their interaction (Table 4). Pelpolage et al. [18] reported that swelling power of potato starch is not influenced by growing location, and water solubility is not affected by variety.

### 2.9. Effects of Variety and Growing Location on Thermal Properties of Starch

**Figure 8:**
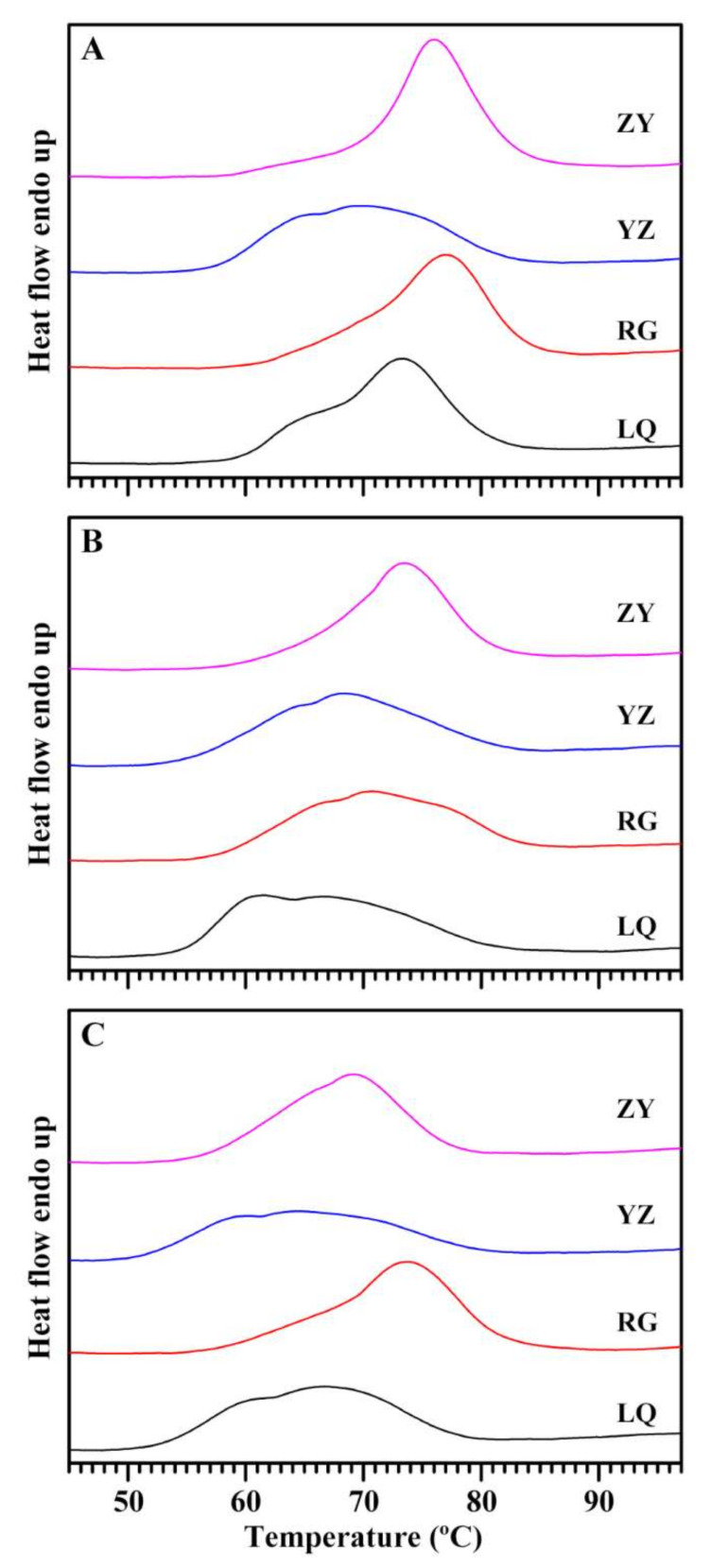
DSC thermograms of starches from root tubers of Ningzishu 1 (**A**), Sushu 16 (**B**), and Sushu 28 (**C**) in growing locations of Linquan (LQ), Rugao (RG), Yangzhou (YZ), and Zunyi (ZY).

Thermal properties provide important reference parameters for evaluating cooking and processing quality of starch. Significant differences in thermograms were detected among different sweet potato varieties and growing locations (Figure 8). Some starches such as starch from Ningzishu 1 grown in ZY exhibited single-peak DSC curve with wide gelatinization temperature range, some starches such as starch from Sushu 28 grown in LQ exhibited two-peak DSC curve, and some starches such as starch from Sushu 16 grown in RG showed three-peak DSC curve (Figure 8). Similar single-, two-, and three-peak DSC curves are detected in sweet potato starches from different varieties [2,9,11]. This complex gelatinization curve is due to that C-type starches have different proportions of A-type crystallinity with high gelatinization temperature and B-type crystallinity with low gelatinization temperature [40,41]. Guo et al. [2] systematically studied thermal properties of sweet potato starch. The single-, two-, and three-peak DSC curves with wide gelatinization temperature range of sweet potato starches can all be fitted into three gelatinization peaks corresponding to the gelatinization of B-, C-, and A-type starch according to gelatinization temperature from low to high. The sweet potato C-type starch consists of heterogeneous granules of A-, B-, and C-type starch. Their different proportions result in single-, two-, and three-peak gelatinization curves in sweet potato starches from different varieties [31]. The different DSC curves of starches from different sweet potato varieties and growing locations indicated that thermal properties of starch were influenced by varieties, growing locations, and their interaction. The gelatinization temperature and enthalpy are presented in Table 4. The gelatinization onset temperature varied from 57.6 °C (YZ) to 62.9 °C (RG), from 54.8 °C (LQ) to 62.8 °C (ZY), and from 51.1 °C (YZ) to 57.9 °C (RG) for Ningzishu 1, Sushu 16, and Sushu 28, respectively. The gelatinization peak temperature varied from 69.5 °C (YZ) to 77.2 °C (RG), from 61.2 °C (LQ) to 73.0 °C (ZY), and from 64.1 °C (YZ) to 73.5 °C (RG) for Ningzishu 1, Sushu 16, and Sushu 28, respectively. The gelatinization conclusion temperature varied from 80.8 °C (LQ) to 84.0 °C (RG), from 80.7 °C (ZY) to 83.0 °C (RG), and from 77.3 °C (ZY) to 81.5 °C (RG) for Ningzishu 1, Sushu 16, and Sushu 28, respectively. The gelatinization temperature range varied from 20.9 °C (LQ) to 24.5 °C (YZ), from 17.9 °C (ZY) to 26.6 °C (YZ), and from 20.9 °C (ZY) to 28.1 °C (YZ) for Ningzishu 1, Sushu 16, and Sushu 28, respectively. The gelatinization enthalpy is the energy requirement for disruption of crystallinity, and ranged from 13.2 J/g (YZ) to 14.3 J/g (LQ), from 12.2 J/g (RG) to 13.3 J/g (ZY), and from 11.7 J/g (YZ) to 13.3 J/g (RG) for Ningzishu 1, Sushu 16, and Sushu 28, respectively. Two-way ANOVA analysis showed that gelatinization temperature and enthalpy of sweet potato were significantly affected by variety, growing location, and their interaction (Table 4). The gelatinization properties of starch are affected by variety, growing location, and their interaction in potato [18] and wheat [16], but only gelatinization onset and peak temperature of starch are affected by varieties in cassava [19].

### 2.10. Effects of Variety and Growing Location on Digestion Properties of Starch

The enzyme hydrolysis of starch can provide important references in estimating starch utilizations in food and nonfood industries. The RDS, SDS, and RS are presented in Table 5. For native starch, Ningzishu 1, Sushu 16, and Sushu 28 had significantly different digestibilities. Three sweet potato varieties exhibited the highest and lowest RDS in growing location of YZ and ZY, respectively, and the highest and lowest RS in ZY and YZ, respectively (Table 5). Abegunde et al. [20] measured the digestion of native starches from 11 sweet potato varieties planted in different places, and found that starches from different cultivars exhibit significantly different digestibilities due to that environmental temperature and soil affect starch development. Guo et al. [2] reported that native starches from nine sweet potato varieties grown in the same location exhibit significantly different digestion properties. In fact, enzyme hydrolysis of native starch is affected by morphology, size, component, amylopectin structure, crystallinity, hydrolysis condition, and type of hydrolyzing enzyme [2,20]. In the present research, two-way ANOVA analysis showed that the digestibility of native starch was significantly influenced by sweet potato variety, growing location, and their interaction (Table 5). For gelatinized starch, the digestion of starch is mainly affected by amylose/amylopectin structure and content [21]. The gelatinized starch had RDS from 70.5% to 81.4%, SDS from 2.1% to 11.0%, and RS from 10.8% to 23.3% among three sweet potato varieties grown in four locations. Two-way ANOVA analysis also showed that the digestibility of gelatinized starch was significantly influenced by sweet potato variety, growing location, and their interaction (Table 5). Tappiban et al. [19] measured the RS of starches from five cassava cultivars grown in two locations, and found that RS of cassava starch is not affected by genotype, environment, and their interaction.

## 3. Materials and Methods

### 3.1. Plant Materials

Three sweet potato varieties of purple-fleshed Ningzishu 1, yellow-fleshed Sushu 16, and white-fleshed Sushu 28 were planted on 27 May and harvested on 31 October 2020. For each variety in one experimental plot, 100 seedlings were planted in five rows with 90 cm between rows and 20 cm between hills and one seedling per hill under routine agronomic practices in four growing locations of Linquan (LQ) [latitude 32°58′32″ N, longitude 115°14′05″ E, altitude 38 m], Rugao (RG) [latitude 32°21′38″ N, longitude 120°26′36″ E, altitude 6 m], Yangzhou (YZ) [latitude 32°23′43″ N, longitude 119°25′46″ E, altitude 8 m], and Zunyi (ZY) [latitude 27°32′05″ N, longitude 107°10′20″ E, altitude 893 m]. All varieties in every growing location were planted repeatedly in three experimental plots. The soil in each growing location was given the same nitrogen treatment of 15 kg/ha applied in the ridge before planting. The fertilizers included urea, superphosphate, and potassium sulfate. Temperature variation of growing location during sweet potato growth stage is presented in Figure 3.

### 3.2. Isolation of Starch from Root Tuber of Sweet Potato

Starch was isolated from fresh root tuber of sweet potato using water homogenization, sieving, and treatment of 0.2% NaOH and anhydrous ethanol following the processes of Guo et al. [2] exactly.

### 3.3. Analysis of Size Distribution of Starch

Starch and water slurry was analyzed with a laser particle size analyzer (Mastersizer 2000, Malvern, Worcestershire, UK) following the processes of Guo et al. [2]. The testing condition included the opacity between 10% and 11% and the mixing at 2000 rpm. The granule size was expressed with volume-weighted mean diameter (D[4,3]).

### 3.4. Analysis of Absorption Spectrum Parameters of Starch–Iodine Complex

Starch was dissolved in dimethyl sulfoxide containing 10% 6.0 M urea (UDMSO) and stained with iodine solution following the processes of Man et al. [22] exactly. The sample was analyzed with a spectrophotometer (BioMate 3S, Thermo Scientific, Chino, CA, USA). The parameters including the absorption value at 550, 620, and 680 nm and the maximum absorption wavelength were obtained from the spectrum.

### 3.5. Determination of Amylose Content

The amylose content (AC) was measured using concanavalin A (Con A) precipitation method with an amylose/amylopectin assay kit (K-AMYL, Megazyme, Bray, Ireland) following its assay procedures. For measuring principle, starch is first dispersed completely into amylose and amylopectin, and separated into two aliquots. The Con A specifically precipitates the amylopectin in an aliquot, and the remaining amylose in the supernatant is quantified. The dispersed amylose and amylopectin in another aliquot are also quantified. The AC is the ratio of amylose to both amylose and amylopectin.

### 3.6. X-ray Diffraction (XRD) Analysis

Starch was analyzed using an XRD spectrometer (D8, Bruker, Karlsruhe, Germany) following the method of Guo et al. [2] exactly. The testing condition included X-ray beam at 40 kV and 40 mA and scanning angle from 3° to 40° 2θ with 0.02° step size. The relative crystalline (RC) was the area percentage of crystalline peaks and total diffraction peak between 4° to 30° 2θ [42].

### 3.7. Fourier Transform Infrared (FTIR) Analysis

Starch was analyzed using a FTIR spectrometer (7000, Varian, Santa Clara, CA, USA) following the method of Guo et al. [2] exactly. The testing condition included 64 scans with 4 cm^−1^ resolution from 4000 to 800 cm^−1^. The spectrum was deconvoluted using Lorentzian line shape with 19 cm^–1^ half-width and 1.9 resolution enhancement factor after baseline correction from 1200 to 900 cm^−1^.

### 3.8. Small Angle X-ray Scattering (SAXS) Analysis

Starch was analyzed using a SAXS system (NanoStar, Bruker, Karlsruhe, Germany) following the method of Cai et al. [43] except that the peak intensity and position were measured with PeakFit V4.12 software. The testing condition included X-ray source with copper rotating anode (0.1 mm filament) at 50 kV and 30 W, cross coupled Göbel mirrors, and 1.5418 Å Cu Kα radiation wavelength.

### 3.9. Rapid Visco Analyzer (RVA) Analysis

Starch was analyzed using a RVA (3D, Newport Scientific, Warriewood, Australia) following the method of Guo et al. [2] exactly. The testing condition included starch (2.5 g) and water (25 mL) slurry, 160 rpm rotating speed, and programmed temperature–time profile with 50 °C for 1 min, from 50 to 95 °C for 3.75 min, 95 °C for 2.5 min, from 95 to 50 °C for 3.75 min, and 50 °C for 1.4 min.

### 3.10. Determination of Swelling Power and Water Solubility

The swelling power and water solubility of starch at 95 °C were measured following the method of Guo et al. [2] exactly. The soluble carbohydrates measured with anthrone-H_2_SO_4_ method were used to evaluate the water solubility (percentage of soluble carbohydrates to total starch), and the swelling power was the ratio of precipitated starch to total starch with subtraction of soluble carbohydrates.

### 3.11. Differential Scanning Calorimetry (DSC) Analysis

Starch was analyzed using a DSC (200-F3, Netzsch, Selb, Germany) following the method of Guo et al. [2] exactly. The testing condition included starch (5 mg) and water (15 μL) sealed in aluminum pan, heating range from 30 to 130 °C, and heating rate at 10 °C/min.

### 3.12. Analysis of Digestion Properties of Starch

The digestibilities of native and gelatinized starches were evaluated using both porcine pancreatic α-amylase (Sigma, A3176, St. Louis, MO, USA) and *Aspergillus niger* amyloglucosidase (Megazyme, E-AMGDF) following the method of Guo et al. [2] exactly. The released glucose was measured with a glucose assay kit (K-GLUC, Megazyme, Bray, Ireland) to convert the degraded starch quantity. The total starch was measured using a total starch assay kit (K-TSTA, Megazyme, Bray, Ireland). The rapidly digestible starch (RDS) and slowly digestible starch (SDS) are the percentage of degraded starch within 20 min and between 20 min and 2 h to the total starch, respectively, and the resistant starch (RS) is the difference between total starch and both RDS and SDS.

### 3.13. Data Analysis

The data difference among different starches with Tukey’s test and the effects of variety, growing location, and their interaction on starch properties with two-way ANOVA test were analyzed with SPSS 19.0.

## 4. Conclusions

Three sweet potato varieties grown in four locations were used to investigate the effects of variety and growing location on starch physicochemical properties in sweet potato. The granule size, iodine absorption parameters and AC of starch were influenced significantly by variety, growing location, and their interaction. C-type starches in root tubers had different proportions of A- and B-type crystallinities in different varieties and growing locations. The ordered degree and lamellar thickness of starch were affected by variety and growing location of sweet potato. The pasting properties, swelling power, water solubility, thermal properties, and digestibility exhibited significant differences among different varieties and growing location. Therefore, in consideration of the applications of starch, it is very important to choose an appropriate variety for planting sweet potato in a specific growing location.

## Figures and Tables

**Table 1:** Effects of variety and growing location on granule size, iodine absorption parameters, and amylose content of starch from sweet potato root tuber.

Variety	Location	D[4,3] (μm)	Iodine Absorption Parameters				AC (%)
			OD620	OD680	OD620/550	λmax (nm)	
Ningzishu 1	LQ	16.06 ± 0.01iB	0.332 ± 0.010bcB	0.297 ± 0.005bB	1.145 ± 0.017abA	606.5 ± 0.5aA	16.4 ± 0.9aA
	RG	18.22 ± 0.02kD	0.339 ± 0.010bcdB	0.302 ± 0.006bcB	1.156 ± 0.033abcA	606.1 ± 0.2aA	16.6 ± 0.3aA
	YZ	13.33 ± 0.01bA	0.275 ± 0.002aA	0.257 ± 0.005aA	1.232 ± 0.021efB	625.6 ± 2.4dB	16.7 ± 0.3aA
	ZY	17.50 ± 0.02jC	0.361 ± 0.005eC	0.321 ± 0.004deC	1.128 ± 0.021aA	606.4 ± 0.4aA	17.7 ± 0.4abA
Sushu 16	LQ	13.79 ± 0.01dB	0.339 ± 0.002bcdB	0.308 ± 0.004bcdAB	1.196 ± 0.006cdeA	613.9 ± 2.6cB	18.4 ± 0.1bcA
	RG	15.39 ± 0.01hD	0.339 ± 0.010bcdB	0.308 ± 0.011bcdAB	1.193 ± 0.021bcdeA	609.3 ± 0.8abA	19.5 ± 0.5cdeA
	YZ	13.53 ± 0.02cA	0.322 ± 0.005bA	0.294 ± 0.007bA	1.208 ± 0.006defA	609.2 ± 2.0abA	19.0 ± 0.4bcdA
	ZY	14.75 ± 0.03gC	0.353 ± 0.003deB	0.326 ± 0.007eB	1.235 ± 0.006efB	610.7 ± 0.4bcAB	21.2 ± 0.2fB
Sushu 28	LQ	14.14 ± 0.01eB	0.334 ± 0.010bcdB	0.302 ± 0.010bcB	1.175 ± 0.015abcdA	611.1 ± 0.6bcB	19.8 ± 0.6cdefAB
	RG	14.19 ± 0.01fC	0.333 ± 0.007bcB	0.298 ± 0.006bB	1.178 ± 0.010bcdA	606.8 ± 1.2aA	21.1 ± 0.3efB
	YZ	12.11 ± 0.01aA	0.260 ± 0.006aA	0.243 ± 0.004aA	1.252 ± 0.026fB	629.0 ± 2.4eC	18.3 ± 0.6bcA
	ZY	14.13 ± 0.02eB	0.347 ± 0.003cdeB	0.316 ± 0.003cdeC	1.191 ± 0.016bcdeA	606.4 ± 0.4aA	20.3 ± 0.3defB
F-value	Variety (2)	94054.64 ***	25.00 ***	29.17 ***	18.10 ***	10.95 ***	109.55 ***
	Location (3)	63443.40 ***	161.64 ***	120.35 ***	19.68 ***	180.67 ***	19.14 ***
	Variety × Location (6)	9144.67 ***	15.41 ***	9.15 ***	7.02 ***	61.03 ***	5.93 **

D[4,3]: volume-weighted mean diameter; OD620 and OD680: absorption value of starch–iodine complex at 620 and 680 nm, respectively; OD620/550: ratio of absorption value of starch–iodine complex at 620 and 550 nm; λmax: maximum absorption wavelength of starch–iodine complex; AC: amylose content. Data are means ± standard deviations, *n* = 3. Values with different lowercase letters in the same column are significantly different (*p* < 0.05), and values with different capital letters in the same column and the same variety are significantly different (*p* < 0.05). The number in the parentheses is the freedom degree, and ** and *** indicates statistically significant effect at 0.01 and 0.001 level, respectively.

**Table 2:** Effects of variety and growing location on relative crystallinity, ordered structure, and lamellar structure parameters of starch from sweet potato root tuber.

Variety	Location	RC (%)	IR Ratio		Lamellar Parameters	
			1045/1022 (cm^−1^)	1022/995 (cm^−1^)	PI (a.u.)	D (nm)
Ningzishu 1	LQ	26.2 ± 0.9aA	0.650 ± 0.002abA	0.974 ± 0.005efC	382.3 ± 2.0cdB	9.72 ± 0.00aA
	RG	25.0 ± 1.3aA	0.699 ± 0.013deB	0.983 ± 0.015fC	418.6 ± 0.6dC	9.80 ± 0.01abcBC
	YZ	25.3 ± 0.7aA	0.709 ± 0.006deB	0.896 ± 0.003aA	357.1 ± 3.0bcA	9.78 ± 0.00abcB
	ZY	24.5 ± 0.5aA	0.676 ± 0.012bcdAB	0.934 ± 0.005bcdB	384.8 ± 4.6cdB	9.82 ± 0.02abcC
Sushu 16	LQ	24.9 ± 1.9aA	0.634 ± 0.001aA	1.103 ± 0.009gB	331.2 ± 1.5bC	9.86 ± 0.03bcA
	RG	22.8 ± 1.7aA	0.659 ± 0.004abcA	0.954 ± 0.010deA	257.7 ± 7.3aA	10.15 ± 0.02eC
	YZ	24.3 ± 0.1aA	0.702 ± 0.012deB	0.945 ± 0.002cdA	289.2 ± 0.8aB	10.21 ± 0.01eC
	ZY	24.9 ± 0.3aA	0.697 ± 0.014deB	0.922 ± 0.014bcA	332.4 ± 4.7bC	10.02 ± 0.01dB
Sushu 28	LQ	23.7 ± 0.4aA	0.678 ± 0.007bcdA	0.977 ± 0.005efC	275.5 ± 8.3aA	9.87 ± 0.08bcA
	RG	26.2 ± 0.8aA	0.695 ± 0.012cdeA	0.925 ± 0.002bcB	417.8 ± 7.2dB	9.76 ± 0.01abA
	YZ	24.7 ± 0.8aA	0.726 ± 0.019eA	0.931 ± 0.003bcdB	332.0 ± 35.1bA	9.88 ± 0.00cA
	ZY	23.4 ± 1.3aA	0.703 ± 0.012deA	0.911 ± 0.003abA	283.2 ± 1.6aA	9.89 ± 0.07cA
F-value	Variety (2)	2.15	13.42 **	73.34 ***	119.16 ***	163.16 ***
	Location (3)	0.46	30.45 ***	197.87 ***	15.42 ***	18.20 ***
	Variety × Location (6)	2.69	3.33 *	52.74 ***	43.11 ***	18.96 ***

RC: relative crystallinity; 1045/1022: ratio of peak intensity at 1045 and 1022 cm^−1^; 1022/995: ratio of peak intensity at 1022 and 995 cm^–1^; PI: lamellar peak intensity; D: lamellar thickness. Data are means ± standard deviations, *n* = 3. Values with different lowercase letters in the same column are significantly different (*p* < 0.05), and values with different capital letters in the same column and the same variety are significantly different (*p* < 0.05). The number in the parentheses is the degree of freedom, and *, ** and *** indicates statistically significant effect at the 0.05, 0.01 and 0.001 level, respectively.

**Table 3:** Effects of variety and growing location on pasting properties of starch from sweet potato root tuber.

Variety	Location	PV (mPa s)	HV (mPa s)	BV (mPa s)	FV (mPa s)	SV (mPa s)	P_Temp_ (°C)	P_Time_ (min)
Ningzishu 1	LQ	4635 ± 29 gC	2546 ± 27eC	2090 ± 29eC	3087 ± 51cB	541 ± 25aA	75.47 ± 0.45eB	4.40 ± 0.07cA
	RG	4171 ± 20bcdA	2478 ± 27dB	1693 ± 45bB	3034 ± 17cB	556 ± 11abA	78.73 ± 0.46gC	4.60 ± 0.07dB
	YZ	4293 ± 65defB	2788 ± 26fD	1504 ± 43aA	3471 ± 42dC	682 ± 16deC	73.08 ± 0.51cA	4.62 ± 0.04dB
	ZY	4273 ± 26cdeB	2171 ± 16aA	2102 ± 10eC	2802 ± 21aA	631 ± 24bcdB	78.98 ± 0.08gC	4.42 ± 0.08cA
Sushu 16	LQ	4656 ± 63gC	2363 ± 42cB	2293 ± 45fD	2925 ± 12bB	562 ± 49abA	71.68 ± 0.03bA	4.42 ± 0.04cB
	RG	4145 ± 34bcA	2306 ± 28bcB	1839 ± 28cA	2938 ± 13bB	633 ± 20bcdAB	74.18 ± 0.03dC	4.60 ± 0.01dC
	YZ	4102 ± 25bA	2113 ± 29aA	1989 ± 7dB	2769 ± 26aA	656 ± 33cdB	72.47 ± 0.03bcB	4.47 ± 0.01cB
	ZY	4383 ± 42efB	2178 ± 16aA	2205 ± 39fC	2947 ± 36bB	769 ± 43fC	76.05 ± 0.48efD	4.22 ± 0.04bA
Sushu 28	LQ	4929 ± 52hD	2338 ± 39bcA	2591 ± 14hC	2942 ± 55bA	604 ± 16abcdA	70.55 ± 0.48aA	4.20 ± 0.07bB
	RG	3918 ± 89aA	2439 ± 26dB	1479 ± 64aA	3026 ± 21cB	587 ± 45abcA	76.88 ± 0.45fC	4.51 ± 0.04cdC
	YZ	4415 ± 79fB	2846 ± 36fC	1569 ± 44aA	3579 ± 35eC	733 ± 32efB	70.07 ± 0.06aA	4.44 ± 0.04cC
	ZY	4739 ± 40gC	2274 ± 22bA	2465 ± 43gB	2920 ± 25bA	645 ± 6 cd A	72.80 ± 0.52cB	4.07 ± 0.07aA
F-value	Variety (2)	42.75 ***	289.82 ***	125.27 ***	173.57 ***	10.11 **	384.62 ***	49.45 ***
	Location (3)	268.63 ***	254.21 ***	787.49 ***	233.50 ***	38.92 ***	377.46 ***	81.60 ***
	Variety × Location (6)	34.44 ***	131.76 ***	98.02 ***	142.61 ***	7.75 ***	50.46 ***	6.17 **

PV: peak viscosity; HV: heat viscosity; BV: breakdown viscosity (PV–HV); FV: final viscosity; SV: setback viscosity (FV–HV); P_Temp_: pasting temperature; P_Time_: peak time. Data are means ± standard deviations, *n* = 3. Values with different lowercase letters in the same column are significantly different (*p* < 0.05), and values with different capital letters in the same column and the same variety are significantly different (*p* < 0.05). The number in the parentheses is the degree of freedom, and ** and *** indicates statistically significant effect at the 0.01 and 0.001 level, respectively.

**Table 4:** Effects of variety and growing location on swelling power, water solubility, and thermal property parameters of starch from sweet potato root tuber.

Varieties	Locations	SP (g/g)	WS (%)	Thermal Property Parameters				
				To (°C)	Tp (°C)	Tc (°C)	ΔT (°C)	ΔH (J/g)
Ningzishu 1	LQ	30.9 ± 0.2cA	12.7 ± 0.5aA	59.9 ± 0.1gB	73.3 ± 0.1fB	80.8 ± 0.1cA	20.9 ± 0.2bA	14.3 ± 0.1cB
	RG	29.9 ± 0.9bcA	12.5 ± 0.6aA	62.9 ± 0.1hC	77.2 ± 0.3gD	84.0 ± 0.5fC	21.1 ± 0.3bA	14.2 ± 0.1cB
	YZ	30.1 ± 0.7bcA	12.9 ± 0.5aA	57.6 ± 0.1fA	69.5 ± 0.6deA	82.1 ± 0.4deB	24.5 ± 0.4cdC	13.2 ± 0.6abcA
	ZY	30.2 ± 0.6bcA	14.4 ± 0.7bcB	60.1 ± 0.1gB	76.2 ± 0.2gC	83.3 ± 0.3efC	23.1 ± 0.2cB	14.1 ± 0.5cB
Sushu 16	LQ	32.6 ± 0.6dB	15.4 ± 0.1cA	54.8 ± 0.2cA	61.2 ± 0.2aA	80.9 ± 0.4cA	26.1 ± 0.3eB	13.2 ± 0.6abcA
	RG	29.9 ± 0.6bcA	15.2 ± 0.8cA	57.7 ± 0.5fC	70.0 ± 0.9eC	83.0 ± 0.3efB	25.3 ± 0.6deB	12.2 ± 0.6abA
	YZ	29.8 ± 0.5bcA	14.2 ± 0.5bcA	55.7 ± 0.4dB	68.7 ± 0.4dB	82.3 ± 0.7deB	26.6 ± 0.9eB	12.7 ± 0.4abcA
	ZY	29.1 ± 0.4bA	17.9 ± 0.6dB	62.8 ± 0.2hD	73.0 ± 0.4fB	80.7 ± 0.5cA	17.9 ± 0.6aA	13.3 ± 0.7bcA
Sushu 28	LQ	28.9 ± 0.2bB	13.5 ± 0.1abA	53.1 ± 0.1bB	66.4 ± 0.5cB	77.4 ± 0.4aA	24.3 ± 0.4cdB	12.8 ± 0.7abcA
	RG	27.4 ± 0.5aA	14.6 ± 0.1bcB	57.9 ± 0.2fD	73.5 ± 0.3fD	81.5 ± 0.3cdC	23.6 ± 0.4cB	13.3 ± 0.7bcA
	YZ	26.7 ± 0.4aA	12.8 ± 0.4aA	51.1 ± 0.3aA	64.1 ± 0.4bA	79.2 ± 0.9bB	28.1 ± 1.1fC	11.7 ± 0.7aA
	ZY	29.8 ± 0.5bcB	15.3 ± 0.6cB	56.5 ± 0.4eC	69.1 ± 0.2deC	77.3 ± 0.2aA	20.9 ± 0.2bA	12.9 ± 0.6abcA
F-value	Variety (2)	62.68 ***	75.16 ***	1467.13 ***	796.78 ***	205.24 ***	41.10 ***	18.39 ***
	Location (3)	23.27 ***	41.71 ***	911.93 ***	644.24 ***	73.36 ***	173.62 ***	5.29 **
	Variety × Location (6)	11.76 ***	4.42 **	195.71 ***	157.12 ***	11.25 ***	62.30 ***	2.78 *

SP: swelling power; WS: water solubility; To: gelatinization onset temperature; Tp: gelatinization peak temperature; Tc: gelatinization conclusion temperature; ΔT: gelatinization temperature range (Tc–To); ΔH: gelatinization enthalpy. Data are means ± standard deviations, *n* = 3. Values with different lowercase letters in the same column are significantly different (*p* < 0.05), and values with different capital letters in the same column and the same variety are significantly different (*p* < 0.05). The number in the parentheses is the degree of freedom, and *, ** and *** indicates statistically significant effect at the 0.05, 0.01, and 0.001 level, respectively.

**Table 5:** Effects of variety and growing location on digestion properties of native and gelatinized starches.

Varieties	Locations	Native Starch			Gelatinized Starch		
		RDS (%)	SDS (%)	RS (%)	RDS (%)	SDS (%)	RS (%)
Ningzishu 1	LQ	3.2 ± 0.1cC	10.3 ± 0.3aB	86.5 ± 0.4gB	70.5 ± 1.1aA	6.2 ± 0.5bcAB	23.3 ± 1.5eB
	RG	2.7 ± 0.1bB	9.2 ± 0.2aA	88.1 ± 0.3ghC	72.9 ± 0.9abcAB	7.9 ± 0.2cdB	19.2 ± 1.1cdA
	YZ	5.1 ± 0.1fD	16.6 ± 0.6cC	78.3 ± 0.7deA	75.0 ± 1.6bcdB	7.7 ± 0.5cdB	17.3 ± 1.4cA
	ZY	2.2 ± 0.1aA	8.8 ± 0.3aA	89.1 ± 0.4hC	74.4 ± 1.8bcdB	5.5 ± 1.6bA	20.1 ± 0.2dA
Sushu 16	LQ	5.0 ± 0.1fC	18.3 ± 0.4dB	76.6 ± 0.5dB	78.4 ± 0.6efB	10.2 ± 0.3efAB	11.5 ± 0.8abA
	RG	4.1 ± 0.1dB	16.3 ± 0.6bcA	79.6 ± 0.7efC	76.0 ± 1.3cdeB	10.7 ± 0.7fB	13.3 ± 0.6bB
	YZ	7.9 ± 0.3hD	29.6 ± 0.9eC	62.5 ± 1.1bA	77.1 ± 0.7deB	11.0 ± 0.4fB	11.8 ± 0.4abA
	ZY	3.3 ± 0.2cA	16.6 ± 0.2cA	80.0 ± 0.4efC	72.6 ± 2.0abA	8.2 ± 1.8cdA	19.2 ± 0.3cdC
Sushu 28	LQ	6.5 ± 0.2gB	19.0 ± 0.6dB	74.5 ± 0.9cB	80.7 ± 0.6fB	8.5 ± 0.3deC	10.8 ± 0.8aA
	RG	4.6 ± 0.0eA	15.1 ± 0.0bcA	80.3 ± 0.1efC	78.3 ± 1.5efA	2.1 ± 0.8aA	19.6 ± 1.1dB
	YZ	10.9 ± 0.3iC	30.9 ± 1.4eC	58.2 ± 1.6aA	81.1 ± 0.3fB	7.9 ± 0.3cdC	11.0 ± 0.5aA
	ZY	4.6 ± 0.1eA	14.7 ± 0.8bA	80.6 ± 0.9fC	81.4 ± 0.2fB	6.7 ± 0.3bcdB	12.0 ± 0.4abA
F-value	Variety (2)	361.59 ***	1267.42 ***	745.02 ***	109.19 ***	75.68 ***	220.52 ***
	Location (3)	125.27 ***	1425.86 ***	727.37 ***	4.77 **	14.92 ***	42.25 ***
	Variety × Location (6)	9.16 ***	88.32 ***	29.75 ***	10.29 ***	19.44 ***	54.79 ***

RDS: rapidly digestible starch degraded within 20 min; SDS: slowly digestible starch degraded between 20 min and 2 h; RS; resistant starch undegraded within 2 h. Data are means ± standard deviations, *n* = 3. Values with different lowercase letters in the same column are significantly different (*p* < 0.05), and values with different capital letters in the same column and the same variety are significantly different (*p* < 0.05). The number in the parentheses is the degree of freedom, and ** and *** indicates statistically significant effect at the 0.01 and 0.001 level, respectively.

## Data Availability

The data are available upon request from the corresponding author.

## Sample Availability

Samples of starches are available from the corresponding author upon reasonable request.
